# Interrelationships among diverse forms of social participation and their associations with basic psychological needs among older adults in Switzerland

**DOI:** 10.3389/fpsyg.2025.1577513

**Published:** 2025-06-06

**Authors:** Shkumbin Gashi, Heidi Kaspar, Martin grosse Holtforth

**Affiliations:** ^1^Department of Health, Bern University of Applied Sciences, Bern, Switzerland; ^2^Department of Clinical Psychology and Psychotherapy, University of Bern, Bern, Switzerland; ^3^Department of Neurology, Psychosomatic Medicine, Inselspital, Bern University Hospital, Bern, Switzerland

**Keywords:** social participation, autonomy, relatedness, competence, basic psychological needs, older adults, interrelations

## Abstract

Our aim was to identify which forms of social participation among older adults are associated with one another and how they relate to the satisfaction of basic psychological needs. Accordingly, we offer insights into the multiple roles older adults take on within their communities and how these roles relate to their wellbeing. We recruited participants for the study using a non-random, multi-pronged recruitment strategy through various channels. A sample of Swiss-based older adults (*N* = 286) completed an online survey containing demographic questions, measures of formal and informal social participation, and the Balanced Measure of Psychological Needs Scale by Sheldon and Hilpert (2012), validated in German by Neubauer and Voss (2016). Our results showed that older adults are active in diverse forms of social participation, highlighting a complex interconnectedness between multiple roles that they assume within their communities. However, the associations between different forms of social participation and the satisfaction of basic psychological needs were nuanced and selective. Through correlational and cluster analyses, we found that informal social participation was associated with balanced psychological needs satisfaction. Furthermore, both excessive and low engagement in certain forms of formal participation were linked to an imbalance in the satisfaction of basic psychological needs. Therefore, our findings inform practice by showing that to better support basic psychological needs satisfaction, informal participation should be prioritized, while formal participation should be personalized and monitored on how it is perceived by older adults, either as overly demanding or insufficiently challenging.

## 1 Introduction

### 1.1 The interconnectedness of social participation in older age

Nowadays, older adults are getting recognized as heterogeneous individuals, defined by their own particular lived experiences, personal characteristics, and social contexts, rather than solely by biological age (Lowsky et al., 2014; Swiss Federal Statistical Office, 2024). Psychologists are emphasizing that older adults have a considerable degree of control over their lives (Diehl et al., 2020), a position supported by evidence showing their direct involvement in decision-making around issues that are relevant to them, such as managing their own health (Wolff and Boyd, 2015). Simultaneously, the sociocultural environment of older adults is likewise continuously altering to accommodate this growing agency, providing more opportunities for them to be socially active. Activities enabled by technological advancements, such as virtual interactions, allow surpassing physical barriers of social participation to a certain extent (Baez et al., 2019), while age-friendly architectural planning (Chau and Jamei, 2021) has made it more appealing for older adults to spend time outside of four walls. Switzerland stands out as a country that facilitates participation in older age through its structures and supportive social context. Concretely, its organizations provide possibilities for older adults to engage voluntarily (i.e., Pro Senectute Schweiz, n.d.), and educational institutions promote lifelong learning (i.e., University of Zurich, n.d.). National reports show that older adults in Switzerland are highly active, be that through volunteering (Lamprecht et al., 2020), physical activities (Swiss Federal Statistical Office, 2023), or outdoor pursuits (Fischer et al., 2021a; Fischer et al., 2021b).

Social participation can range from less demanding endeavors like sharing a common hobby to more complex ones like community activism (Levasseur et al., 2010). These endeavors are often extended outside one’s home, involving people beyond nuclear family members. Therefore, social participation is a direct way for people to engage with their surrounding communities. The concept is highlighted by both international stakeholders (i.e., World Health Organization, 2020) and Swiss governance (i.e., Stadt Zürich, 2020) as a central aspect of aging well in today’s society.

How older adults engage in their communities, interact with others, and assume roles varies greatly. Some engage in new activities post-retirement, while others remain active in roles that they held in earlier phases of life. Some, however, withdraw from their community roles, either voluntarily or due to health or other relevant life events. For those who stay active, social participation takes many forms, allowing them to find and assume roles that suit them. This variety of options is very significant, as older adults differ in the resources, time, and energy they dedicate to social participation activities (Van Hees et al., 2020). Such variety is supported not only by the range of available participation opportunities but also by the interconnected nature of these activities, allowing older adults to engage in self-selection processes that combine their diverse social interests with their individual capacities and resources.

This self-selection process has been documented in the literature that has shown how engagement in one form of social participation overlaps with engagement in others, as evidenced below. Hank and Stuck (2008) conducted a cross-sectional study with more than 27,000 participants (50 and older) across Europe and examined the associations between voluntary work, informal caregiving, and helping people inside family circles and others in the community. Specifically, their results showed that those older adults who engaged in voluntary activities were also likely to be involved in caregiving roles and in helping others. Another cross-sectional study conducted in Turkey by Yılmaz et al. (2023) found associations between various forms of engagement and social interactions. The study had a sample of 1,224 participants over the age of 60 and had an aim to compare the frequency of participation and differences between rural and urban contexts. The authors also found that the participants from urban areas who had phone calls with others also attended diverse cultural activities and pursued gardening, pointing to rich interactions and social roles. In Germany, Bukov et al. (2002), using longitudinal data from the Berlin Aging Study (BASE), found that older adults involved in political activities were also active in collective and productive activities. In Switzerland, Dawson-Townsend (2019) used data from the Swiss Household Panel for the years 2013 and 2016 to examine social participation among adults 60 years and older. The author employed cluster analysis to identify patterns of participation and showed that while some older adults are likely to be active, specifically in informal, non-demanding interactions with family and non-kin, others are both active in such contexts and in more demanding ones through club membership and volunteering.

Despite the findings listed above, the evidence on the interrelationships between different forms of social participation is inconclusive. Informal forms of social participation, such as nature pursuits, home gatherings, etc., are often overlooked in research examining the co-occurrence of participation types. At the same time, formal participation is frequently treated too broadly and grouped under general categories such as organizational involvement, with limited attention to the diversity within specific forms of formal participation.

We aimed to address this gap in literature by examining the correlations among 13 forms of social participation in Switzerland. These ranged from more formal forms, such as involvement in political, religious, and leisure organizations or caring communities, to more informal activities, like social gatherings at home, nature pursuits, sport activities, etc. (presented in detail in Table 1).

**Table 1:** Examples of formal and informal social participation.

Formal participation	Informal participation
Sport organization	Nature Pursuits (i.e., walking, hiking, biking, etc.)
Cultural organization	Restaurants/Cafés
Religious organization	Home Gatherings (at one’s own or someone else’s home or garden, i.e., eating, grilling, having an apéro, etc.)
Political organization	Sports Events (i.e., attending as a spectator, participating, or contributing to organization)
Humanitarian organization (i.e., Red Cross).	Theater/Cinema/Concerts (i.e., attending as a spectator, participating, or contributing to organization)
Leisure organization (i.e., card games, choir, reading circle)	Museums/Zoos
Caring communities (i.e., visiting services, neighborhood assistance).	—

This table categorizes formal and informal forms of social participation based on the structure and context in which they occur.

By including both formal and informal forms of participation, we aimed to expand the understanding of how these forms interact and co-occur in older adults’ social lives. For this, we tested the hypothesis:

*H1*: Older adults who engage in one form of social participation are likely to engage in other forms of social participation, too.

To further add knowledge into the complex nature of the social participation of older adults, we examined the frequency of participation in different forms of social participation and the membership rates in organizations through the research question:

RQ1: How frequently do older adults participate in 13 forms of social participation, and what are their rates of active and passive memberships in organizations and associations?

### 1.2 The associations between diverse forms of social participation and satisfaction of basic psychological needs

One common characteristic of the literature on social participation is that its associations with health and wellbeing are mostly studied from a loss-oriented perspective. This is best evidenced in a bibliometric analysis by Fu et al. (2021), which examined more than 7,000 articles about social participation. When examining the occurrence of the top keywords of these articles, more general keywords such as “elderly,” “aging,” and “aged” appeared most often, followed by keywords such as “disability,” “quality of life,” “depression,” “dementia,” “cognitive impairment,” “mortality,” and” frailty.” Furthermore, this analysis also showed that research has recently (2017–2019) focused on loneliness, cognitive impairments, and functional decline, further confirming a predominantly medical and decline-oriented focus in studies on social participation. However, the World Health Organization (WHO) is advocating for more focus on evidence on older age beyond the medical lenses. Its recent global initiative on aging, “The Decade of Healthy Aging 2021–2030,” is built around the premise that older adults’ wellbeing should be defined by their holistic functioning as individuals rather than just by the narrowed criteria of the presence or absence of a disease or illness. As part of this approach, WHO calls for research that investigates and highlights the abilities, intrinsic capacities, and social environments of older adults (2020). Thus, through our study, we respond to the WHO’s call for additional research on health beyond the medical perspective by examining the associations between social participation and the satisfaction of basic psychological needs: relatedness, competence, and autonomy, deriving from self-determination theory (Ryan and Deci, 2018). We argue that our study aligns with the WHO’s holistic approach to older age by examining the associations between relatedness, competence, and autonomy as representations of intrinsic capacities and social participation as representative of the social environment. The satisfaction of basic psychological needs is presumed to promote psychological growth and wellbeing, while their dissatisfaction/frustration is thought to undermine the above (Ryan and Deci, 2018). The self-determination theory defines relatedness as the human need for emotional closeness to others, competence as the need to attribute change in one’s own life to personal abilities, and autonomy as the need for those changes to result from personal choices and preferences (Ryan and Deci, 2018). Previous studies have provided substantial evidence on the positive relationships between satisfaction of these three basic needs and various aspects of health and wellbeing, thereby establishing the significance of these constructs for optimal functioning. For example, Martela et al. (2022) conducted a large-scale survey with more than 48,000 participants from 27 European countries and found positive associations between the satisfaction of the three needs with place in society, life satisfaction, happiness, and meaning in life, as well as negative associations with depression. A study by Stenling et al. (2021) also confirmed the negative associations between needs satisfaction and depressive symptoms. Furthermore, research conducted in cross-cultural (Tang et al., 2021) and cross-generational (Lataster et al., 2022) settings has shown that the three needs have universal applicability and relevance over diverse cohorts. Martela and Ryan (2023) also suggested that satisfaction of basic psychological needs should be included in population surveys on wellbeing. Specifically, the authors argue that autonomy, competence, and relatedness can be integrated within the concept of eudaimonic wellbeing and, combined with experiential and evaluative indicators, can provide a more complete picture of people’s overall life quality.

In literature on older adults, particular relevance is placed on physical activities and exercises when looking at how social participation relates to the satisfaction of basic psychological needs. For example, Solberg et al. (2013) conducted a 16-week randomized controlled trial with a Norwegian sample of older adults (n = 118) in which 99 participants were assigned to the experimental group and exercised three times per week for a duration of 13 weeks, while 39 participants were assigned to the control group, received no exercise treatment, and continued with their usual daily routine. The perceived competence was higher in the experimental group after the intervention. In another study, Kirkland et al. (2011) compared exercisers and non-exercisers among 209 community-based older adults in the United States and found that exercisers reported higher satisfaction of the three basic psychological needs as well as higher intrinsic and both self-determined and non-self determined external motivation. Jones et al. (2019) studied the motivations related to exercise and basic psychological needs satisfaction among 160 United States rural-dwelling older adults comprising three groups: structured exercisers, unstructured exercisers, and non-exercisers. The authors found that structured exercisers had higher satisfaction of competence and relatedness but lower autonomy satisfaction compared to unstructured exercisers. One specific cross-sectional study that aligns with our aim is the research paper by Chen and Zhang (2021). The authors included a wide range of social participation forms and activities relevant for the Chinese context, including organizations in which people help their neighbors organize ceremonies such as weddings or funerals, hobbies, neighborhood communities that support dispute resolutions, organizations managing cleaning, traffic, and other relevant issues for the well-functioning of communities. Their sample consisted of 1,458 Chinese adults, aged 60 years and older. The authors found that the satisfaction of relatedness, competence, and autonomy all correlated positively with community participation. In our study, we expand upon the findings of Chen and Zhang (2021) by examining the correlations between 13 forms of social participation and the satisfaction of basic psychological needs in a sample of Swiss older adults. By focusing on a western context, our research provides relevant evidence from a cultural setting that is quite different from the study realized in China by Chen and Zhang (2021).

To examine potential associations between basic psychological need satisfactions and social participation, we tested one hypothesis along with six sub-hypotheses:

*H2*: Social participation correlates with satisfaction of basic psychological needs.

*H2.1*: High social participation correlates positively with relatedness satisfaction.

*H2.2*: High social participation correlates negatively with relatedness dissatisfaction.

*H2.3*: High social participation correlates positively with autonomy satisfaction.

*H2.4*: High social participation correlates negatively with autonomy dissatisfaction.

*H2.5*: High social participation correlates positively with competence satisfaction.

*H2.6*: High social participation correlates negatively with competence dissatisfaction.

Additionally, to further expand the evidence on the complex relationships between social participation and the satisfaction of basic psychological needs, we aimed to identify distinct profiles that emerge from the interaction between these two concepts.

To this end, we formulated the following exploratory research question:

RQ2: What types of profiles emerge from the interaction between social participation and the satisfaction/dissatisfaction of basic psychological needs?

We also examined older adults’ level of satisfaction and dissatisfaction of basic psychological needs with two aims: first, to compare results with existing evidence in the literature, and second, to better contextualize the interrelationships of basic psychological needs with social participation. For this purpose, we formulated the research question:

RQ3: What degree of satisfaction and dissatisfaction do older adults report across the dimensions of relatedness, competence, and autonomy?

## 2 Methodology

### 2.1 Participants

Of the 286 participants who completed our survey, 64% were female and 36% were male. All were aged 65 years and older: 46.9% were between 65–70 years, 29.7% between 71–75, 18.5% between 76–80, and 4.9% were 81 years and older. More than half (57%) lived with their partners, while 40% lived alone. The detailed sample characteristics are listed in Table 2.

**Table 2:** Sociodemographic characteristics of participants.

Sociodemographic characteristics	*n*	%
Gender		
Female	183	64.0
Male	103	36.0
Age		
65–70	134	46,9
71–75	85	29,7
76–80	53	18,5
Over 80	14	4.9
Citizenship		
Swiss Citizenship	252	88.1
Dual Citizenship (Swiss and other)	22	7.7
Non-Swiss Citizenship	10	3.5
Missing	2	0.7
Civil status		
Married	145	50.7
I live with my partner in a cohabiting relationship.	26	9.1
I live separately from my partner.	6	2.1
I am single.	22	7.7
I am divorced.	47	16.4
I am widowed.	34	11.9
No answer	6	2.1
Household		
I live alone.	115	40.2
I live together with my spouse/life partner.	163	57.0
I live together with my child/ren.	2	0.7
I live together with other relatives.	2	0.7
I live with other people.	3	1.0
No answer	1	0.3
Education		
Mandatory Schooling (up to 9th grade)	7	2.4
Vocational Training, Apprenticeship	98	34.3
Secondary School (Maturity School, Vocational Maturity, Diploma Secondary School, etc.)	28	9.8
Higher Technical School	62	21.7
University (University, University of Applied Sciences, etc.)	91	31.8
Financial situation		
I manage very well	107	37.4
I manage well	120	42.0
I manage just right	32	11.2
I have to restrict myself	20	7.0
I have to restrict myself a lot.	5	0.7
No answer	2	0.7

### 2.2 Data collection

We began data collection after receiving approval from the Ethics Commission of the Faculty of Human Sciences at the University of Bern, Switzerland. Employing a non-random, multi-pronged recruitment strategy, we promoted our survey through several channels: Facebook, Zurich Seniors University, a + Swiss Platform Ageing Society, Loopings.ch, Crossiety app, and seniors portal-schweiz.ch. We developed an online survey in German using ILIAS (ILIAS open source e-Learning e.V.), a platform used by the University of Bern. To ensure the methodological rigor and contextual compatibility of the survey, we implemented a multi-step process. First, we compiled the survey based on the instruments presented below in the methods section. Afterwards, we conducted a pre-test phase with the Commission for Old Age, a local advisory body comprising six older adults and a political representative from the local municipality. This step was particularly useful in evaluating the comprehensibility, cognitive load, and contextual appropriateness of the items in the survey for older adults in Switzerland. Each member of the commission (excluding the representative of the municipality) first completed a pen-and-paper survey and then participated in a joint meeting with the first author of the study, where they presented their feedback and recommendations, which we incorporated into the survey. In addition to the forms of social participation adapted from a Dutch study (Van Groenou and Deeg, 2010), the commission proposed modifications and additions to forms of social participation to better reflect the Swiss context. For example, when we formulated the item “leisure organization,” we integrated several sub-activities, such as card games, choir, reading circle, etc.” These options reflect activities that are common among older adults in Switzerland. Card games, in the survey are listed as “Jassen,” which is popular in Switzerland, particularly among older adults (see Table 1 above for a list of all forms of participation included in the study). Another instance of adjustments made to the survey based on the feedback from the Commission for Old Age relates to the scaling. Specifically, to examine the basic psychological needs satisfaction, we used the German version (Neubauer and Voss, 2016) of the Balanced Measure of the Psychological Needs Scale (Sheldon and Hilpert, 2012). The German version of the scale includes a 7-point Likert scale response format, which five out of six older adults in the commission found it cognitively demanding. Therefore, we applied the 5-point Likert response format, consistent with the original version of the scale by Sheldon and Hilpert (2012). Despite this adjustment, the scale demonstrated satisfactory internal consistency, as seen below in the instrument’s alpha values. We then uploaded the survey to the ILIAS platform and consulted two of the six older adults mentioned above regarding the online version’s accessibility. Together with the first author, they went through the survey step-by-step during a joint meeting. After completing all these steps, we started with the distribution of the online survey to the target population. Participants accessed the survey via a link that could be opened on any device (PC, laptop, tablet, or mobile phone). The first page provided instructions, participant rights, and information on our ethical guidelines and efforts to protect their integrity as participants. We did not require any identifiable information, such as home addresses or names. However, for those wishing to receive the study results once published, we included an option to provide their email addresses at the end of the survey. Out of the 348 respondents, 286 were included in the analysis based on exclusion criteria, which omitted participants under the age of 65 and those who did not complete the sections on social participation and the Balanced Measure of Basic Psychological Needs Scale.

### 2.3 Instruments

To capture the demographic characteristics of participants in our study, we relied on a survey by Grillenberger (2016), used in an assessment of aging experiences in Germany. Thirteen items obtained aspects such as participants’ age, gender, household constellation, education level, civil status, and other demographic information. All response options had a multiple-choice closed format, except for the one about citizenship, which had an open format.

To assess social participation, we adopted questions from a survey by Van Groenou and Deeg (2010), originally used in a study on social participation in the Netherlands. This survey contained questions about social participation, ranging from engagement and memberships in various organizations to informal pursuits such as sports, culture, and other casual activities like eating out in restaurants, visiting zoos, museums, etc. Our survey, informed both by Van Groenou and Deeg (2010) items and by the feedback from the Commission for Older Age (as described above in procedure), contained 15 items for measuring social participation. In the first two items we asked participants, “Are you a passive (item 1) or active (item 2) member of any organization?,” with “yes” and “no” as response options. Through 15 other items, we measured the frequency of participation in concrete formal and informal social participation forms.

Formal participation included participation in sports, cultural, religious, political, humanitarian, and leisure organizations, as well as caring communities. Informal participation included more casual, often self-organized and concrete activities, such as nature pursuits, dining at restaurants or cafes, home gatherings, sports events, etc. (Table 1). Answers were organized on a five-option Likert scale, varying from daily, weekly, at least once a month, at least once a year, and never.

We used the Balanced Measure of the Psychological Needs Scale of Sheldon and Hilpert (2012), validated in German by Neubauer and Voss (2016), to measure the satisfaction and dissatisfaction of three basic psychological needs: autonomy, competence, and relatedness. Response options consisted of a 5-point Likert scale ranging from “never” to “always” as proposed by Sheldon and Hilpert (2012). The scale has 18 items: relatedness satisfaction (I1, I3, I5), relatedness dissatisfaction (I2, I4, I6), competence satisfaction (I7, I9, I11), competence dissatisfaction (I8, I10, I12), autonomy satisfaction (I13, I15, I17), and autonomy dissatisfaction (I14, I16, I18). We computed Cronbach’s alpha to assess the internal consistency of this scale for each of its six sub-scales, and the results were satisfactory across all of them: relatedness satisfaction (*α* = 0.759), relatedness dissatisfaction (*α* = 0.708), competence satisfaction (*α* = 0.755), competence dissatisfaction (α = 0.715), autonomy satisfaction (*α* = 0.742), and autonomy dissatisfaction (*α* = 0.804).

### 2.4 Data analysis

We used IBM SPSS v. 27 (IBM Corporation, Armonk, NY, United States) to carry out the analysis on the data collected for our study. First, we cleaned the data and prepared them for analysis. Afterwards, we performed a Cronbach’s alpha (α) reliability test for the six subscales of the Balanced Measure of the Basic Psychological Needs Scale. We used descriptive statistics such as frequencies, means, minimums, maximums, and standard deviations to obtain an overview of the trends in our sample. Next, we analyzed associations between various forms of social participation and correlations of those forms with basic psychological needs using the Spearman-Rho correlation because we treated both sets of variables as ordinal. Finally, we conducted a K-means cluster analysis to determine how participants of our study can be grouped based on both their frequency of social participation and basic psychological needs satisfaction/dissatisfaction.

## 3 Results

### 3.1 Patterns and rates of social participation

Our findings indicate that older adults are highly active in organizations and associations, with active membership (70.6%) exceeding passive membership rates (62.9%). In the first form, older adults actively engage with the organization(s), whereas through passive membership, they stay informed about the activities without being directly involved in them. These results are presented in detail in Table 3.

**Table 3:** Active and passive participation in organizations and associations.

Type of participation	Response	Frequency	Percent	Valid percent	Cumulative percent
Active	Yes	203	70.6	70.6	70.6
	No	83	29.0	29,0	99.7
	Total	286	100.0	100.0	
Passive	Yes	180	62.9	62.9	62.9
	No	96	33.6	33.6	96.5
	Total	286	100.0	100.0	

Apart from the general analysis of memberships in organizations/associations, we also conducted an in-depth analysis of the frequency of participation in diverse forms of social participation. Sports organizations had the highest participation rates, with 34.6% of the participants reporting weekly involvement. Cultural organizations also showed high participation rates, with 14.7% participating weekly and 36.4% monthly. Moreover, monthly participation rates for caring communities were 19.6 and 23.4% for leisure organizations. Regarding informal participation activities, the highest levels were observed in nature pursuits, with 46.2% of older adults participating daily and 43.4% weekly. Visits to restaurants or cafes were also common, with 44.8% visiting them weekly and 39.2% monthly. Cultural activities also had high participation rates, as 49.3% were active in theaters, cinemas, or concerts weekly, and 52.1% visited museums or zoos monthly. When focusing on “never” responses, formal participation forms showed the highest rates, for example, political organizations (52.4%), humanitarian associations (49.3%), and caring communities (43.7%). Among informal forms of social participation, only participation in sports events had a notably high “never” response rate (33.2%). All other forms of informal participation had low levels of non-participation. More details are shown in Table 4.

**Table 4:** Participation frequencies in diverse forms of social participation.

Type of participation	Form	Frequency of participation				
		Daily	Weekly	At least once a month	At least once a year	Never
Formal	Sport organization	1.7%	34.6%	8.4%	7.0%	41.3%
	Cultural organization	0.3%	14.7%	36.4%	24.1%	21.0%
	Religious organization	6.3%	10.1%	18.2%	57.3%	8.0%
	Political organization	0.7%	2.4%	10.5%	26.6%	52.4%
	Humanitarian organization	0.7%	8.7%	11.9%	21.3%	49.3%
	Leisure organization (i.e., card games, choir, reading circles).	0.7%	15.7%	23.4%	14.3%	37.4%
	Caring Communities (i.e., visiting services, neighborhood assistance).	2.8%	14.3%	19.6%	9.1%	43.7%
Informal	Nature pursuits (i.e., walking, hiking, biking, etc.)	46.2%	43.4%	6.6%	3.1%	0.7%
	Restaurants/Cafés	3.5%	44.8%	39.2%	10.5%	2.1%
	Home Gatherings (at one’s own or someone) else’s home or garden (i.e., eating, grilling, having an apéro, etc.)	2.8%	27.3%	49.7%	17.1%	3.1%
	Sports Events (i.e., attending as a spectator, participating, or contributing to organization)	1.7%	14.3%	23.8%	26.9%	33.2%
	Theater/Cinema/Concerts (i.e., attending as a spectator, participating, or contributing to organization)	9.4%	49.3%	36.4%	4.9%	0.0%
	Museums/Zoos	2.8%	34.6%	52.1%	10.5%	0.0%

The percentages represent the share of respondents reporting their frequency of participants in each specific form of social participation. Percentages may not sum to 100% due to rounding or missing responses. “Missing” includes participants who skipped the question or selected “no answer”.

### 3.2 Satisfaction of basic psychological needs scores

Scores on the Balanced Measure of Basic Psychological Needs Scale showed two notable trends. First, the participants scored high on the satisfaction of all three basic needs: relatedness, competence, and autonomy (mean = 13.085, 12.270, 11.339), and moderately on their dissatisfaction/frustration (mean = 7.162, 7.329, 7.360). Second, results showed higher variability in answers for dissatisfaction of the three needs (Std. deviation = 2.878, 2.805, 3.211) compared to their satisfaction (Std. deviation = 1.954, 2.347, 2.356). More details are shown in Table 5.

**Table 5:** Degree of satisfaction and dissatisfaction of three basic psychological needs.

Need	*N*	Minimum	Maximum	Mean	Std.-deviation
Relatedness satisfaction	282	5.00	15.00	13.085	1.954
Relatedness dissatisfaction	283	3.00	15.00	7.162	2.878
Competence satisfaction	277	3.00	15.00	12.270	2.347
Competence dissatisfaction	276	4.00	15.00	7.329	2.805
Autonomy satisfaction	277	4.00	15.00	11.339	2.356
Autonomy dissatisfaction	275	3.00	15.00	7.360	3.211

### 3.3 Correlations between diverse forms of social participation

To interpret the correlations, we applied the Cohen (1988) three-level effect size criteria, small (*r* = 0.10), medium (*r* = 0.30), and large (*r* = 0.50), alongside *p*-values, which assessed the significance of those correlations. We found significant correlations, ranging from small to large, between 13 forms of social participation. For example, engagement in sports organizations showed small but significant correlations with cultural, religious, and political organizations. Similarly, cultural organizations showed small correlations with religious and humanitarian organizations, while religious organizations also had small correlations with participation in leisure and sports organizations. Political organizations also showed small correlations with nature pursuits and, cultural events (theater, cinema, or concerts, etc.) Moreover, informal activities such as home gatherings showed small associations with caring communities, and cultural activities (theater/cinema/concerts). More significant associations, meaning medium-level correlations, were noticed in several cases, for example, between humanitarian organizations and caring communities or leisure organizations, while leisure organizations also correlated moderately with political organizations. The largest correlation was observed between informal activities like visiting zoos or museums and being active in theater, cinema, or concerts. In addition to the significant correlations detected in our study, several other correlations between forms of social participation were not significant (*p* > 0.05). For example, participation in sports organizations did not significantly correlate with nature pursuits, restaurants/cafes, or home gatherings. Similarly, involvement in religious organizations did not show meaningful correlations with cultural activities such as engagement in theater, cinema, or informal sports events. All correlations, both significant and nonsignificant, are shown in Table 6.

**Table 6:** Correlations between different forms of social participation.

First variable	Second variable	*r* values
Sport organization	Culture organization	*r* = 0.236**
Sport organization	Religious organization	*r* = 0.143*
Sport organization	Political organization	*r* = 0.136*
Sport organization	Humanitarian organization	*r* = 0.173**
Sport organization	Leisure organization	*r* = 0.153*
Sport organization	Caring communities	*r* = 0.136*
Sport organization	Informal sport events	*r* = 0.277**
Sport organization	Theater/Cinema/Concerts	*r* = 0.212**
Sport organization	Nature Pursuits	*r* = 0.059
Sport organization	Restaurants/Cafes	*r* = 0.095
Sport organization	Home gatherings	*r* = 0.053
Sport organization	Zoos/Museums	*r* = 0.106
Culture organization	Sport organization	*r* = 0.236**
Culture organization	Religious organization	*r* = 0.220**
Culture organization	Political organization	*r* = 0.136*
Culture organization	Humanitarian organization	*r* = 0.297**
Culture organization	Leisure organization	*r* = 0.344**
Culture organization	Caring communities	*r* = 0.233**
Culture organization	Restaurants/Cafes	*r* = 0.140*
Culture organization	Home gatherings	*r* = 0.139*
Culture organization	Informal sport events	*r* = 0.135*
Culture organization	Theater/Cinema/Concerts	*r* = 0.384**
Culture organization	Zoos/Museums	*r* = 0.299**
Culture organization	Nature pursuits	*r* = 0.046
Religious organization	Sport organization	*r* = 0.143*
Religious organization	Culture organization	*r* = 0.220**
Religious organization	Political organization	*r* = 0.249**
Religious organization	Humanitarian organization	*r* = 0.206**
Religious organization	Leisure organization	*r* = 0.206**
Religious organization	Caring communities	*r* = 0.319**
Religious organization	Restaurants/Cafes	*r* = 0.121*
Religious organization	Nature pursuits	*r* = 0.101
Religious organization	Home gatherings	*r* = 0.115
Religious organization	Informal sport events	*r* = 0.078
Religious organization	Theater/Cinema/Concerts	r =. 077
Religious organization	Zoos/Museums	r =. -043
Political organization	Sport organization	*r* = 0.136*
Political organization	Culture organization	*r* = 0.232**
Political organization	Religious organization	*r* = 0.249**
Political organization	Humanitarian organization	*r* = 0.277**
Political organization	Leisure organization	*r* = 0.302**
Political organization	Caring communities	*r* = 0.264**
Political organization	Nature pursuits	*r* = 0.125*
Political organization	Restaurants/Cafes	*r* = 0.141*
Political organization	Theater/Cinema/Concerts	*r* = 0.230**
Political organization	Home gatherings	*r* = 0.070
Political organization	Informal sport events	*r* = 0.104
Political organization	Zoos/Museums	*r* = 0.104
Humanitarian organization	Sport organization	*r* = 0.173**
Humanitarian organization	Culture organization	*r* = 0.297**
Humanitarian organization	Religious organization	*r* = 0.206**
Humanitarian organization	Political organization	*r* = 0.277**
Humanitarian organization	Leisure organization	*r* = 0.321**
Humanitarian organization	Caring communities	*r* = 0.432**
Humanitarian organization	Home gatherings	*r* = 0.157*
Humanitarian organization	Informal sport events	*r* = 0.194**
Humanitarian organization	Theater/Cinema/Concerts	*r* = 0.172**
Humanitarian organization	Zoos/Museums	*r* = 0.137*
Humanitarian organization	Nature pursuits	*r* = 0.061
Humanitarian organization	Restaurants/Cafes	*r* = 0.043
Leisure organization	Sport organization	*r* = 0.153*
Leisure organization	Culture organization	*r* = 0.343**
Leisure organization	Religious organization	*r* = 0.240**
Leisure organization	Political organization	*r* = 0.302**
Leisure organization	Humanitarian organization	*r* = 0.321**
Leisure organization	Caring communities	*r* = 0.262**
Leisure organization	Restaurants/Cafes	*r* = 0.195**
Leisure organization	Theater/Cinema/Concerts	*r* = 0.151*
Leisure organization	Nature pursuits	*r* = 0.063
Leisure organization	Home gatherings	*r* = 0.056
Leisure organization	Informal sport events	*r* = 0.120
Leisure organization	Zoos/Museums	*r* = 0.109
Caring communities	Sport organization	*r* = 0.136*
Caring communities	Culture organization	*r* = 0.233**
Caring communities	Religious organization	*r* = 0.319**
Caring communities	Political organization	*r* = 0.264**
Caring communities	Humanitarian organization	*r* = 0.432**
Caring communities	Leisure organization	*r* = 0.262**
Caring communities	Home gatherings	*r* = 0.186**
Caring communities	Nature pursuits	*r* = 0.039
Caring communities	Restaurants/Cafes	*r* = –0.025
Caring communities	Informal sport events	*r* = 0.057
Caring communities	Theater/Cinema/Concerts	*r* = 0.086
Caring communities	Zoos/Museums	*r* = 0.083
Nature pursuits	Political organization	*r* = 0.125*
Nature pursuits	Theater/Cinema/Concerts	*r* = 0.124*
Nature pursuits	Sport organization	*r* = 0.059
Nature pursuits	Culture organization	*r* = 0.046
Nature pursuits	Religious organization	*r* = 0.101
Nature pursuits	Humanitarian organization	*r* = 0.061
Nature pursuits	Leisure organization	*r* = 0.063
Nature pursuits	Caring communities	*r* = 0.039
Nature pursuits	Restaurants/Cafes	*r* = 0.101
Nature pursuits	Home gatherings	*r* = 0.086
Nature pursuits	Informal sport events	*r* = −0.014
Nature pursuits	Zoos/Museums	*r* = 0.016
Restaurants/Cafes	Culture organization	*r* = 0.140*
Restaurants/Cafes	Religious organization	*r* = 0.121*
Restaurants/Cafes	Leisure organization	*r* = 0.195**
Restaurants/Cafes	Political organization	*r* = 0.141*
Restaurants/Cafes	Home gatherings	*r* = 0.230**
Restaurants/Cafes	Theater/Cinema/Concerts	*r* = 0.212**
Restaurants/Cafes	Zoos/Museums	*r* = 0.199**
Restaurants/Cafes	Sport organization	*r* = 0.095
Restaurants/Cafes	Humanitarian organization	*r* = 0.043
Restaurants/Cafes	Caring communities	*r* = −0.025
Restaurants/Cafes	Nature pursuits	*r* = −101
Restaurants/Cafes	Informal sport events	*r* = 0.095
Home gatherings	Culture organization	*r* = 0.139*
Home gatherings	Humanitarian organization	*r* = 0.157*
Home gatherings	Caring communities	*r* = 0.186**
Home gatherings	Restaurants/Cafes	*r* = 0.230**
Home gatherings	Informal Sport Events	*r* = 0.183**
Home gatherings	Theater/Cinema/Concerts	*r* = 0.239**
Home gatherings	Zoos/Museums	*r* = 0.222**
Home gatherings	Sport organization	*r* = 0.053
Home gatherings	Religious organization	*r* = 0.115
Home gatherings	Political organization	*r* = 0.070
Home gatherings	Leissure organization	*r* = 0.056
Home gatherings	Nature pursuits	*r* = 0.086
Informal sport events	Sport organization	*r* = 0.277**
Informal sport events	Culture organization	*r* = 0.135*
Informal sport events	Humanitarian organization	*r* = 0.194**
Informal sport events	Home gatherings	*r* = 0.183**
Informal sport events	Theater/Cinema/Concerts	*r* = 0.177*
Informal sport events	Zoos/Museums	*r* = 0.128*
Informal sport events	Religious organization	*r* = 0.078
Informal sport events	Political organization	*r* = 0.104
Informal sport events	Leisure organization	*r* = 0.120
Informal sport events	Caring communities	*r* = 0.057
Informal sport events	Nature pursuits	*r* = −0.014
Informal sport events	Restaurants/Cafes	*r* = 0.095
Theater/Cinema/Concerts	Sport organization	*r* = 0.212**
Theater/Cinema/Concerts	Culture organization	*r* = 0.384**
Theater/Cinema/Concerts	Political organization	*r* = 0.230**
Theater/Cinema/Concerts	Humanitarian organization	*r* = 0.172**
Theater/Cinema/Concerts	Leisure organization	*r* = 0.151*
Theater/Cinema/Concerts	Home gatherings	*r* = 0.239**
Theater/Cinema/Concerts	Restaurants/Cafes	*r* = 0.212**
Theater/Cinema/Concerts	Informal sport events	*r* = 0.177*
Theater/Cinema/Concerts	Zoos/Museums	*r* = 0.500**
Theater/Cinema/Concerts	Religious organization	*r* = 0.077
Theater/Cinema/Concerts	Caring communities	*r* = 0.086
Zoos/Museums	Culture organization	*r* = 0.299**
Zoos/Museums	Humanitarian organization	*r* = 0.137*
Zoos/Museums	Restaurants/Cafes	*r* = 0.199**
Zoos/Museums	Home gatherings	*r* = 0.222**
Zoos/Museums	Informal sport events	*r* = 0.128*
Zoos/Museums	Theater/Cinema/Concerts	*r* = 0.500**
Zoos/Museums	Sport organization	*r* = 0.106
Zoos/Museums	Religious organization	*r* = –0.043
Zoos/Museums	Political organization	*r* = 0.104
Zoos/Museums	Leisure organization	*r* = 0.109
Zoos/Museums	Caring communities	*r* = 0.083
Zoos/Museums	Nature pursuits	*r* = 0.016

*p* < 0.01 (two-tailed) significant at the 0.01 level (**); *p* < 0.05 (two-tailed) significant at the 0.05 level (*). Correlations with no asterisks (*r* values without * or **) are not statistically significant (*p* > 0.05). The *r* values indicate the strength and direction of the relationship between variables.

### 3.4 Correlations between basic psychological needs satisfaction and social participation

Our findings showed several significant correlations between diverse forms of social participation and basic psychological needs satisfaction and dissatisfaction. Relatedness satisfaction correlated positively with caring communities and home gatherings, while competence satisfaction correlated with home gatherings. Autonomy satisfaction was also positively associated with home gatherings, nature pursuits, and engagement in cinemas/concerts/theaters. On the other hand, autonomy dissatisfaction correlated negatively with visits to restaurants and cafes, but neither relatedness nor competence dissatisfaction showed any significant correlations with any of the 13 forms of social participation examined in our study. Based on the three-level Cohen (1988) effect size criteria, all correlations had small effect sizes. Informal social participation activities dominated in variables that correlated with the basic psychological needs satisfaction/dissatisfaction, with the exception of caring communities, which represents a form of formal social participation (see Table 7 for details).

**Table 7:** Significant correlations between basic psychological needs and social participation forms.

Basic psychological need	Social participation form	*r* Values
Relatedness satisfaction	Home Gatherings (at one’s own or someone) else’s home or garden (i.e., eating, grilling, having an apéro, etc.)	*r* = 0.187**
Relatedness satisfaction	Caring Communities (i.e., visiting services, neighborhood assistance).	*r* = 0.171**
Competence satisfaction	Home Gatherings (at one’s own or someone) else’s home or garden (i.e., eating, grilling, having an apéro, etc.)	*r* = 0.162**
Autonomy satisfaction	Nature Pursuits (i.e., walking, hiking, biking, etc.)	*r* = 0.132*
Autonomy satisfaction	Home Gatherings (at one’s own or someone) else’s home or garden (i.e., eating, grilling, having an apéro, etc.)	*r* = 0.184**
Autonomy satisfaction	Theater/Cinema/Concerts (i.e., attending as a spectator, participating, or contributing to organization)	*r* = 0.124*
Autonomy dissatisfaction	Restuarants/Cafes	*r* = −0.166**

The correlation is significant at the 0.01** level (two-sided). The correlation is significant at the 0.05*level (two-sided). The correlation is calculated through Spearman-Rho because both sets of variables are treated as ordinal.

### 3.5 A cluster analysis on social participation and satisfaction of basic psychological needs

To address the research question, *what types of profiles emerge from the interrelation between social participation and satisfaction/dissatisfaction of basic psychological needs*?, we conducted a k-means cluster analysis. The aim was to group participants of our study based on the combination of their frequency of social participation and their scores on the basic psychological needs. Out of the entire sample, 196 participants were included in this analysis. ^1^We transformed the variables into z-scores to account for variations in the two instruments that measured social participation and basic psychological needs. To determine the optimal clusters for the k-means analysis, we first calculated the within-cluster sum of squares (WSS) for various solutions (k2-k6) through the formula:


WSS=Error Mean SquarexDegrees of Freedom.

**Figure 1:**
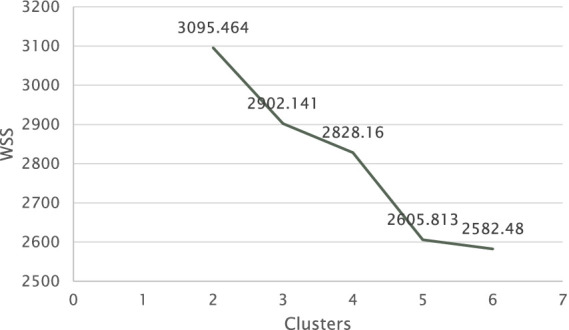
Scree plot showing the within-cluster sum of squares (WSS) for different numbers of clusters. The WSS values are calculated based on *z*-scores of the data.

**Figure 2:**
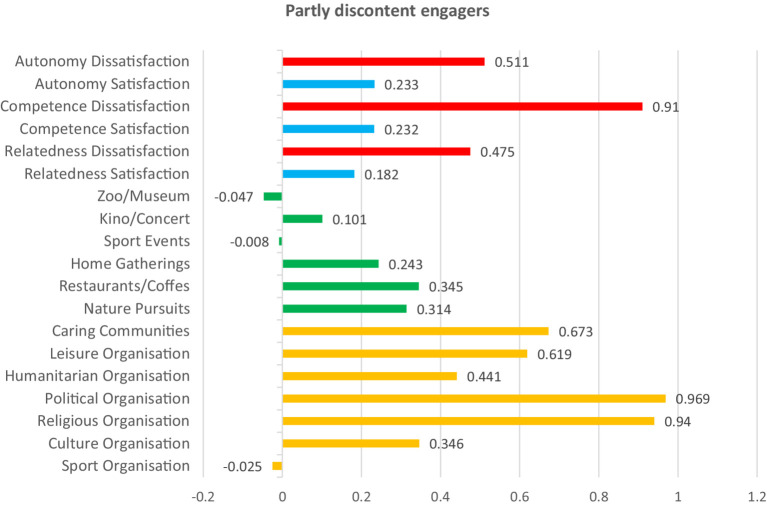
Basic needs satisfaction and social participation among partly discontent engagers. Red bars indicate dissatisfaction of basic needs. Blue bars indicate satisfaction of basic needs. Green bars represent non-formal social participation. Orange bars represent formal social participation.

**Figure 3:**
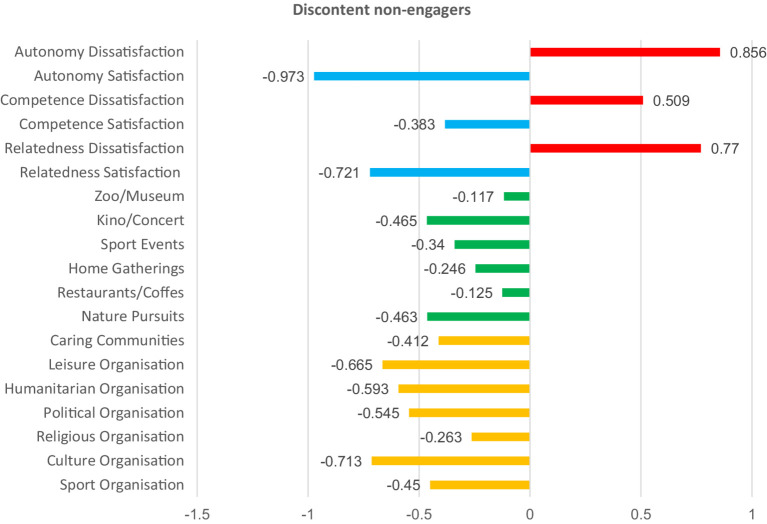
Basic needs satisfaction and social participation among discontent non-engagers. Red bars indicate dissatisfaction of basic needs. Blue bars indicate satisfaction of basic needs. Green bars represent non-formal social participation. Orange bars represent formal social participation.

**Figure 4:**
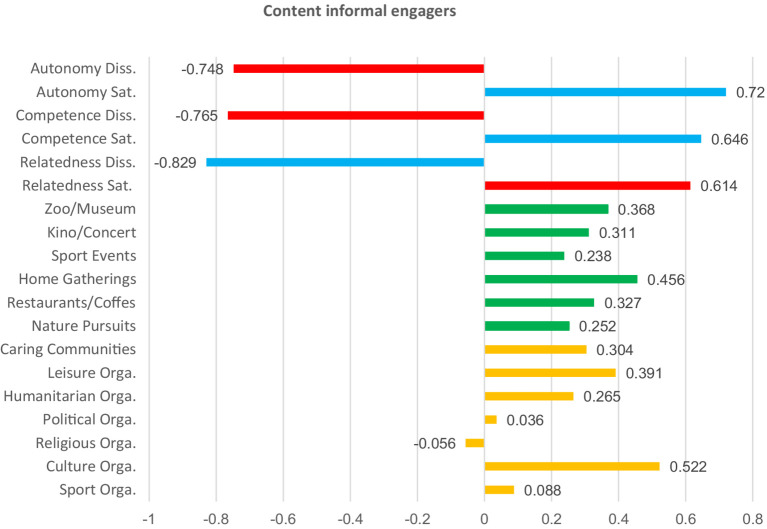
Basic needs satisfaction and social participation among content informal engagers. Red bars indicate dissatisfaction of basic needs. Blue bars indicate satisfaction of basic needs. Green bars represent non-formal social participation. Orange bars represent formal social participation.

**Figure 5:**
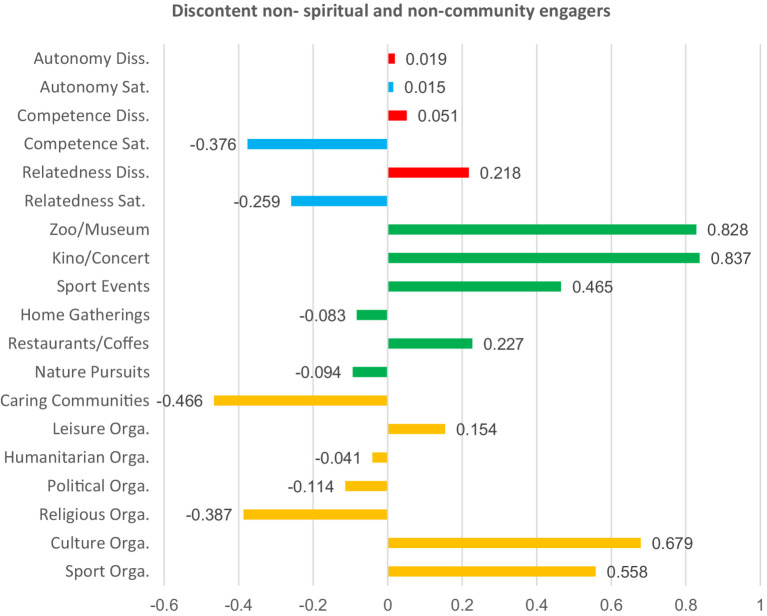
Basic needs satisfaction and social participation among discontent non-spiritual and non-community engagers. Red bars indicate dissatisfaction of basic needs. Blue bars indicate satisfaction of basic needs. Green bars represent non-formal social participation. Orange bars represent formal social participation.

**Figure 6:**
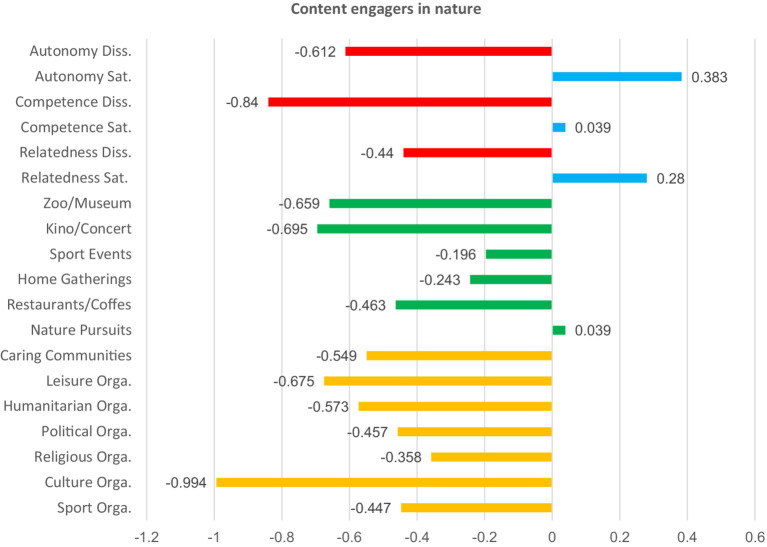
Basic needs satisfaction and social participation among content engagers in nature. Red bars indicate dissatisfaction of basic needs. Blue bars indicate satisfaction of basic needs. Green bars represent non-formal social participation. Orange bars represent formal social participation.

Afterwards, we summed the total of the within-cluster sum of squares for all variables across all solutions, and based on those gained values, we visualized the results (Figure 1) as the final step in determining the optimal clusters for analysis. We created a scatter plot in Excel and applied the “elbow method” to determine the number of optimal clusters. As visualized below in Figure 1, the optimal number of clusters was five, because that is where the “elbow” is seen, as the difference between k5 and k6 is small, indicating non-marginal differences between clusters afterwards. The five clusters of participants had relatively uniform sizes (*N* = 34, 37, 49, 38, 38). The results of the k-means cluster analysis are presented numerically in Table 8 and visualized in Figures 2–6.

**Table 8:** Final cluster centers for K-means analysis.

Variable	Final cluster centers					Anova (Sig.)
	Partly discontent engagers (1)	Discontent non-engagers (2)	Content informal engagers (3)	Discontent non-spiritual and non-community engagers (4)	Content engagers in nature (5)	
Sport Orga.	−0.025	−0.450	0.088	0.558	−0.447	<0.001
Culture Orga.	0.346	−0.713	0.522	0.679	−0.994	<0.001
Religious Orga.	0.940	−0.263	−0.056	−0.387	−0.358	<0.001
Political Orga.	0.969	−0.545	0.036	−0.114	−0.457	<0.001
Humanitarian Orga.	0.441	−0.593	0.265	−0.041	−0.573	<0.001
Leisure Orga.	0.619	−0.665	0.391	0.154	−0.675	<0.001
Caring Communities	0.673	−0.412	0.304	−0.466	−0.549	<0.001
Nature Pursuits	0.314	−0.463	0.252	−0.094	0.039	0.002
Restaurants/Coffes	0.345	−0.125	0.327	0.227	−0.463	<0.001
Home Gatherings	0.243	−0.246	0.456	−0.083	−0.243	0.001
Sport Events	−0.008	−0.340	0.238	0.465	−0.196	0.002
Theater/Kino/Concert	0.101	−0.465	0.311	0.837	−0.695	<0.001
Zoo/Museum	−0.047	−0.117	0.368	0.828	−0.659	<0.001
Relatedness Sat.	0.182	−0.721	0.614	−0.259	0.280	<0.001
Relatedness Diss.	0.475	0.770	−0.829	0.218	−0.440	<0.001
Competence Sat.	0.232	−0.383	0.646	−0.376	0.039	<0.001
Competence Diss.	0.910	0.509	−0.765	0.051	−0.840	<0.001
Autonomy Sat.	0.233	−0.973	0.720	0.015	0.383	<0.001
Autonomy Diss.	0.511	0.856	−0.748	0.019	−0.612	<0.001

To describe and differentiate the five clusters, we assigned labels that best reflect the characteristics of their members. We labeled these clusters based on the dominant patterns observed across variables representing forms of social participation and basic psychological needs satisfaction. “Partly discontent engagers” scored high in almost all forms of social participation, particularly in formal ones such as religious and political organizations. Their satisfaction with relatedness, competence, and autonomy was slightly above the mean, yet they simultaneously reported moderate to high levels of dissatisfaction of those basic psychological needs. “Discontent non-engagers” reported below-average participation across all social participation forms. Satisfaction with basic psychological needs was low, while dissatisfaction was high across the three needs. “Content informal engagers” scored high in almost all forms of social participation, with a particular emphasis on informal activities. Here, participants scored high in the satisfaction of all three basic psychological needs, and low in their dissatisfaction. “Discontent non-spiritual and non-community engagers” scored high in both formal and informal forms of social participation. However, several low scores were noted in spiritually and solidarity-based forms of social participation, such as religious, humanitarian, and caring communities. Participants scored high in the satisfaction of autonomy, and in the dissatisfaction of the three needs, but low in the satisfaction of competence and relatedness. Last, “content engagers in nature “showed participation levels slightly above the mean for nature-related activities but lower than the mean in all other forms of social participation. These participants also reported balanced needs, characterized by high satisfaction and low dissatisfaction across all three needs. The differences between all clusters are significant (see Table 8 and Figures 2–6 for details).

## 4 Discussion

Older adults who participated in our study were very active in their communities and engaged in a wide range of both formal and informal activities. This is consistent with and supports previous Swiss data. According to the Swiss Federal Statistical Office (2022), in 2020, people aged 65–74 years provided the second-highest rate of informal voluntary work, slightly behind people aged 55–64 years old, compared to all other populations (15 years and older). Previous data has also shown that older adults in Switzerland are highly engaged in other forms of social participation beyond voluntary work, especially in activities that influence their wellbeing. Their levels of physical activity are comparable to those of younger individuals (Swiss Federal Statistical Office, 2023), with high engagement in outdoor pursuits, including activities like cycling (Fischer et al., 2021a) and hiking (Fischer et al., 2021b). Our findings align with the sources highlighted above because nature pursuits had the highest participation rates in our study, with 46.2% of participants reporting engaging in nature pursuits (i.e., walking, hiking, biking, etc.) daily. In our study, the most prevalent forms of formal social participation were engagement in sports, culture, and religious organizations, reflecting general trends in Switzerland reported by other sources. Specifically, the 2020 Swiss volunteering survey by Lamprecht et al. (2020) also found that older adults in Switzerland most frequently engaged in church activities, as well as cultural and sports events.

When we interpret the main trends in social participation in our study, as reported in Tables 3, 4, three patterns emerge. First, participants reported slightly higher active membership in organizations than passive membership. We interpret this as a preference for more direct forms of involvement, which offer richer opportunities for social interactions. At the same time, although on lower frequencies than active ones, the presence of passive memberships points to selective strategies, where older adults may choose to maintain minimal interactions with certain organizations without investing time or resources. This combination between active and passive membership allows older adults to balance their social activity and commitment in line with personal capacities and needs.

The second pattern is that engagement in informal participation was more prevalent than in formal participation. This indicates a broader tendency among older adults to favor flexible and less demanding forms of participation. Informal activities are easier to adapt to individual routines, abilities, and wishes. Hence, they have the advantage of allowing meaningful interaction without the administrative or time-related obligations that formal setting participation requires. The third pattern is that among formal social participation, engagement in sport organizations was the most prevalent one. This, we argue, may very likely be due to the combination of benefits these activities provide, such as physical activity, regular social contact, and structured routines. Furthermore, participating in sports organizations, for many older adults, is a continuation of long-established interests and hobbies, providing a familiar way to remain active. Taken together, these three patterns show how older adults’ social participation choices align with personal preferences, available resources, and the value they place in different levels and forms of social participation.

Moving on to another finding in our study, older adults reported high levels of satisfaction and moderate dissatisfaction of basic psychological needs (relatedness, competence, and autonomy). These results align with the current reality of older adults in Switzerland, who are not only living longer but also healthier and possess agency, as shown in the latest publication of the Swiss Federal Statistical Office (2024). According to the “Panorama Swiss Society 2024,” older adults of today are more active, more mobile, and feel younger, challenging the stereotypical view of them as frail.

The high levels of basic psychological needs satisfaction confirm this finding. In other studies (i.e., Yang et al., 2021), older adults report even higher levels of basic psychological needs satisfaction than other populations, such as college students. When looking at the general trends about the satisfaction of three needs in our study, we observed a hierarchical satisfaction, with relatedness ranking highest, followed by competence and autonomy satisfaction. This suggests that older adults in our study felt a stronger sense of connection with their social environment but felt less personal mastery or control over their own lives. The comparatively lower satisfaction of competence and autonomy indicates that the satisfaction of those two needs may be less than optimal. However, this does not necessarily point to a shortfall. This may also be a desire for more opportunities to experience themselves as self-directed and capable, hence reflecting a motivational drive, a desire for more competence and autonomy.

Relatedness being the most satisfied basic need in our study may be partially explained by specific contextual factors, such as the high levels of social engagement among Swiss older adults, highlighted in both other sources mentioned above, as well as the reported trends in social participation in our study. Other studies also show that contextual and cultural factors play a significant role in influencing which need is prioritized. For example, in a cross-cultural study, Tang et al. (2021) found that older adults in China reported higher levels of competence satisfaction, while French older adults reported greater autonomy satisfaction. Arguments about why, particularly competence, appears higher among Chinese older adults may be found in the long-standing traditions and values such as those rooted in Confucianism, which tend to view aging not as a decline but rather as a culmination of lifelong learning and moral development (Nie, 2021). Moreover, this may also be attributed to the traditional values of community organization forms in the Chinese context, where authority and the ability to competently fulfill one’s role within its social contexts are essential to sustaining one’s position in society (Fei, 1947/1992). In contrast, the higher autonomy satisfaction among French older adults can be attributed to the country’s cultural values that prioritize personal freedom and individualism (i.e., Liu, 2024).

Another central result in our study relates to the hypothesis that older adults who engaged in one form of social participation were likely to be active in other forms of social participation. Our results, as illustrated in Table 6, confirmed this hypothesis because they showed multiple significant correlations observed between 13 forms of social participation, both formal and informal. This is consistent with previous research showing that older adults often take on diverse roles within their communities and combine them. For example, prior studies have demonstrated that older adults who volunteer are also likely to engage in caregiving (Hank and Stuck, 2008) or political activities (Bukov et al., 2002). Similarly, Yılmaz et al. (2023) found that older adults who garden are also likely to attend concerts and maintain social connections. Dawson-Townsend (2019) further showed that while some older adults in Switzerland are primarily involved in informal interactions only, such as spending time with their children or meeting friends, others engage in these informal activities and also participate in clubs and volunteer in their communities. Our results support Dawson-Townsend’s (2019) conclusion that Swiss older adults are active in their communities, engaging in both informal and formal activities. However, we provide a more detailed analysis by showing co-occurrences across 13 specific forms of social participation, both formal and informal. While the Dawson-Towsend’s study, employs broader social participation categories such as, volunteering, club memberships, meeting friends, and visiting children, we build upon them and make even more detailed distinctions. We differentiate between engagement in various organizations, including religious, political, leisure, etc. Furthermore, we also capture very specific and informal social participation activities such as nature pursuits, sports events, home gatherings, etc. In general, our findings show that older adults often assume multiple, potentially beneficial, and motivating roles in their communities. For instance, informal activities like nature pursuits or going out to restaurants and cafes are related to personal wellbeing, whereas formal social participation, such as humanitarian or political involvement, tends to benefit both the individual and the collective.

Concretely, correlations between informal and formal activities, i.e., nature pursuits and engagement in political organizations, show that older adults combine roles that are related to their wellbeing with those influencing community. This supports the World Health Organization (2020) policy focus in “The Decade of Healthy Aging 2020–2030,” which portrays older adults as people with agency for their own wellbeing and for society. Although multiple correlations between forms of participation were significant, they varied in effect sizes. For example, individuals involved in leisure organizations were moderately likely to be active in political and cultural organizations, while those participating in caring communities were also moderately likely to be involved in humanitarian or religious organizations. One correlation with a high effect size was observed between involvement in one category of cultural activities, such as theatres, cinemas, concerts with another one, such as visiting zoos and museums. When focusing the interpretation on medium to high correlations, we notice three trends. First, there are older adults who engage across multiple areas, whether in recreational or civic spaces. Second, there are those committed to spirituality and solidarity, such as those active in caring communities and humanitarian or religious organizations, who seem to prioritize collective wellbeing. Finally, there are older adults who strongly prioritize cultural engagement through involvement in theaters, cinemas, and concerts, and visiting zoos and museums. While our study does not allow us to draw conclusions about the internal and external motivations of older adults or about causal relationships, it does, however, provide some patterns that reflect different orientations and approaches toward social participation in older age. First, the individuals who engaged in civic and recreational forms of social participation pointed to a broad, cross-domain orientation. Their participation is not limited to a single sphere, but it connects personal enjoyment and community involvement. This shows a form of social openness and willingness to act both for oneself and others, making their social engagement both a private good and a public act. Second, the correlations among participation in caring communities, religious and humanitarian activities highlight a participation logic based on care, solidarity, and social commitment. Here participation becomes about sustained forms of social connectedness and resonates with the moral orientation that places value on care and responsibility for others. Third, the associations between cultural activities illustrate a different form of participation, which is more receptive compared to other productive forms, nonetheless rich in meaning. The focus on these activities reflects a desire to remain in contact with public culture and shared imagination. Another aspect of these results that merits attention is the non-significant correlations between social participation forms detected in our study. These should not be overlooked, as they suggest that, alongside the interconnected forms of social participation, some operate independently from one another. In summary, these findings challenge the idea that participation can be universally enhanced through generic interventions, instead they show that those interventions should recognize and respond to the plurality of participation logics.

Another central result from our study involved examining the correlations between 13 forms of social participation and the satisfaction or dissatisfaction of the three basic psychological needs through six sub-hypotheses. Each of them addressed both the satisfaction and dissatisfaction of relatedness, competence, and autonomy in relation to social participation. Our results showed a partial confirmation of these hypotheses, as we observed statistically significant correlations only for some associations with small effect sizes according to Cohen’s (1988) criteria. In practice, this suggests that while social participation was correlated beyond chance with the satisfaction of basic psychological needs from a statistical perspective, how satisfied these needs are and how frequently older adults engage in them likely depends on factors above and beyond the ones captured in our study. Generally, the significant results confirm and align with the body of evidence that shows that there are notable associations between wellbeing and social participation (i.e., Kleiner et al., 2022; Niedzwiedz et al., 2016, etc.). The low strength of those correlations, however, aligns with a critique by Dawson-Townsend (2019) about cross-sectional studies on social participation and health, arguing that such studies often amplify the strength of these associations. In the introduction section, we highlighted the 2018 study of Chen and Zhang conducted in China because it had a similar aim as our study. Our findings also support their findings, because, similar to our research, their correlations between social participation and the satisfaction of basic psychological needs were significant but showed small effect sizes. Focusing on satisfaction/dissatisfaction of specific needs that are associated with social participation, we notice two main trends. First, autonomy satisfaction/dissatisfaction showed the most significant correlations (4 instances), followed by relatedness satisfaction (2 instances) and competence satisfaction (1 instance). Second, most correlations were linked to informal activities like home gatherings, restaurants/cafes, or nature pursuits. Since correlational analyses do not justify causal conclusions, it remains unclear whether older adults with higher autonomy satisfaction are more drawn to informal activities or if participating in these activities fosters a stronger sense of autonomy. It might also be that satisfaction of basic psychological needs, particularly autonomy and informal forms of social participation, mutually reinforce each other, offering complementary benefits for older adults. However, more research is needed to examine the interaction of these variables over time.

To further group participants, we performed a k-means cluster analysis on the basis of their levels of need satisfaction and engagement in diverse forms of social participation. The analysis identified five groups of participants, highlighting key distinctions and trends within them. Individuals either involved in nature-based activities or those predominantly in informal social participation activities demonstrated higher levels of satisfaction and low levels of dissatisfaction across basic psychological needs, performing best of all groups. Other participants who engaged both formally and informally at the same time also showed noticeable patterns. For example, those who were more active in religious and political organizations than in other forms reported unbalanced needs satisfaction. The same was true for those who participated less in religious, humanitarian, and caring communities compared to other forms. The fact that participation in religious organizations is associated with unbalanced needs satisfaction both when it is higher or lower than other forms suggests that, in many cases, it is not just about increasing participation in a straightforward manner but rather about finding a balanced, moderate level of participation. Last, participants with generally low social participation scored low in satisfaction of the basic psychological needs and high in their dissatisfaction, compared to four other groups, which had higher overall engagement. The k-means results further challenge the reductive assumption that among older adults, social participation is predictable, linear, and homogenous. Rather than a straightforward increase in wellbeing with higher participation in all cases, our results show a complex, non-linear relationship between the form, frequency, and context of participation and the satisfaction of basic psychological needs. This provides support to the idea that, besides quantity, it is also the quality and configuration of social participation that influence its associations with the satisfaction of autonomy, competence, and relatedness. This brings us to the conclusion that the interactions between social participation and satisfaction of basic psychological needs are complex, non-linear, and context-contingent, which has both implications for future research and practice, explained in more detail in the section below.

## 5 Limitations, recommendations for research and implications for practice

Our study has its limitations that should be taken into account when interpreting the results. First, the non-random recruitment may have introduced selection bias, having direct effects on the generalizability of our findings. Second, the survey was only available online and in German, which has limited participation to individuals with internet access and fluency in the language. This may have excluded a portion of the target population, such as older adults with low digital literacy or different linguistic backgrounds in Switzerland. Furthermore, participants in our study were physically autonomous, as they were active in one or more organizations and displayed high scores on the satisfaction of basic psychological needs. Therefore, these results are highly unlikely to be generalized to people who may have health issues that disable them from being active in communities or are isolated for any reason whatsoever. Moreover, only 4.9% of the sample were 81 years and older, which means they are underrepresented here. We also want to acknowledge that we did not apply corrections such as False Discovery Rate (FDR) or Bonferroni for multiple comparisons of correlations between different forms of social participation. While we are aware that this increases the potential for Type I errors, we focused on avoiding Type II errors, because the specific hypothesis about social participation interrelationships was exploratory in nature. However, we recommend considering this trade-off when interpreting our results and also in future research, particularly when confirmatory hypotheses are tested.

Despite these limitations, our results are consistent with existing evidence in the literature and provide valuable insights for both future research and for policymakers aiming to enhance social participation among older adults. Our results, most particularly the k-means analysis, generate questions for future research regarding the non-linear and complex associations between social participation and basic psychological needs satisfaction. For instance, why do nature-based and informal activities relate to higher levels of autonomy, competence, and relatedness? Is it because these activities are inherently better suited to the psychosocial context of aging because they allow individuals to be active on their own terms, free from structural expectations and rigid roles, or are there other factors that explain these differences? On the other hand, what are the structural or interpersonal factors in formalized settings of religious or political organizations that explain imbalanced need satisfaction profiles? Do these reflect role strain, unmet expectations, a desire for more personal agency, or perhaps a tension between individual psychological needs and collective identity?

Potential practical implications of our results are just as important. First, we recommend that municipalities and relevant institutions promote accessible green spaces and support community initiatives that enable informal social activities such as walking groups, bike tours, community gardens, open cultural events, etc. This recommendation is supported by our results which have identified significant relationships between the satisfaction of basic psychological needs and nature-based social participation, and although we cannot make any assumptions for causal interactions, we can statistically conclude that between the two there is a mutually reinforcing dynamic. Older adults with high satisfaction of basic psychological needs may find such environments attractive to engage in because they are active in activities that simultaneously support their social interactions and health outcomes. In turn, being in such rewarding settings, they are likely to contribute and be part of activities in more stable and sustainable ways, hence positively influencing more robust social participation activities.

A second recommendation which concerns formal social participation, emphasizes the need for individualized regular tracking of social engagement, particularly within organizations, to prevent both overburdening and underchallenging, which may be both influenced by and influence the satisfaction of basic psychological needs. And this is significant because putting people in inflexible or overwhelming environments may have associations with unbalanced needs satisfaction, hence risking turning social participation into a source of strain rather than a rewarding and enjoyable experience.

## Data Availability

The original contributions presented in the study are included in the article, further inquiries can be directed to the corresponding author/s.

## Footnotes

^1^For the cluster analysis, only participants with complete data across all participation items were included (N = 196 out of 286). SPSS automatically excluded cases with missing values on any variable used in the analysis (listwise deletion).
